# Affections of the Nervous System Dependant on Diseases of the Permanent Teeth

**Published:** 1868-11

**Authors:** 


### SELECTED ARTICLES.

### ARTICLE VIII.

### *Affections of the Nervous System Dependent on Diseases of the Permanent Teeth.*

(Continued.)

V.—*Chronic trismus from impaction of lower dens sapientiæ.*—In a man aged twenty-three, with large teeth and comparatively small maxillary bones, the lower wisdom teeth were imbedded, and unable, from want of room, to come into place. The result was recurrent pain and swelling within the mouth, followed by a sudden attack of "lock-jaw," apparently caused by contraction of the left masseter muscle, which, after four months. duration, was cured by extraction of the left second molar, the wisdom tooth being out of reach. The posterior fang of the extracted tooth was much eroded by absorption.

VI.— *Wry-neck from carious teeth of lower jaw.*—A young woman whose head had, for more than six months, been drawn down nearly to the left shoulder, with considerable pain, was relieved in a few days by the removal of a stump and a partially decayed tooth from the left side of the lower jaw.

VII.—*Epilepsy from a carious tooth.*—A boy, agəd thirteen, under the care of Dr. Ramskill, had frequent attacks of epilepsy, occurring about seven or eight o'clock in the evening. Examination detected "a molar tooth considerably decayed, with a swollen gum around it, and partly growing over into the cavity." It was not very tender to the touch, nor did the examination give rise to toothache. The extraction of this tooth was followed by cessation of the fits.

VIII.—*Tetanus from mechanical irritateon of the pulp.*—The case of a gentleman, (quoted from Tome's Dental Surgery,) who, having broken off a front tooth, went immediately to a prominent dentist in Paris and had an artificial crown pivoted with a gold peg upon the fang. After severe pain for four or five days, trismus set in and was soon followed by tetanus and death.

IX.—*Neuralgia of neck and arm from carious molar.*—Dr. Hyde Salter, the brother of the writer, had suffered much from attacks of inflammation in the left lower anterior molar, which was extensively excavated by caries. At the age of seventeen these acute symptoms had ceased for two or three years, leaving nothing but "an occasional grumbling uneasiness in it." At this time neuralgicpains began to extend from the tooth down into the neck in the left side, and thence over the collar bone down the left arm; these pains enduring for several days and then remitting. There was no actual pain in the tooth itself, nor any tenderness in it, nor in the adjacent gum, nor any appearance of inflammation." "The situation of the pain in the neck, and clavicular and supra-mammary regions, was exactly that of the descending cutaneous branches of the cervical plexus, and the part of the arm where the aching was the most intense and intolerable was at the insertion of the deltoid." These symptoms disappeared with the extraction of the offending tooth and have never since returned.

X.—*Paralysis of the arm from an impacted and carious wisdom tooth.*—Miss B—, aet. twenty-four, had suffered for a fortnight from paralysis of the left arm, accompanied by continuous rheumatic pain in the whole limb, arising from a carious left lower wisdom tooth, which had pierced the gum, but was placed low down and horizontally, the crown pressing forward against the second molar. The extraction of this tooth instantly releived the arm-symptoms. In the operation the inferior maxillary nerve was injured, "so that the teeth on the left side of the lower jaw, and the integument of the lip around the region of the mental foramen, were numbed. This, however, passed away in a few days."

XI.—*Neuralgia of face, neck and arm, with partial paralysis of the latter, from carious wisdom tooth.*—Miss W—"was suffering from constant aching pain in the left side of the face and neck and in the left arm. The pain sometimes became intensely severe. The arm had lost nearly all muscular power." These symptoms, after resisting all medical treatment for two years, disappeared in a few hours after the removal of the tooth.

XII.—*Amaurosis caused by crowding of teeth.*—In this case, (reported by Mr. Hancotk in the *Lancet* of 1859, p. 80,) a boy, aged eleven, whose sight had been previously unimpaired, found upon waking one morning that he was entirely blind. About a month afterwards he was admitted to Charing-Cross Hospital, where it was discovered that his teeth were "much crowded and wedged together; the jaw, in fact, not being large enough for them." Accordingly two permanent and four milk molar teeth were extracted, and "on the same evening the boy could distinguish light from darkness, and on the following morning could make out objects. From this time his sight rapidly improved, and he was dismissed cured on the 28th, (eleven days after,) the only treatment beyond the removal of the teeth being two doses of aperient medicine.

"Dr. Watson, (Lectures on Physic, fourth ed., vol. ii, p. 351,) mentions a very similar case. But the blindness was confined to one eye; it recurred two or three times, and was on each occasion cured by tooth-extraction."

XIII.—*Deafness from carious teeth.*—Mr. Cattlin reports the case of a lady who had for about three months suffered acute pains in a diseased right lower molar, and in the corresponding ear and side of the neck, and who had been deaf for four days. "The inflamed tooth was extracted, and hearing returned within an hour after the operation."

XIV.—*Perverted nutrition from dental nervous irritation*—Under this heading three cases are quoted from Mr. Hilton's work ("On the Influence of Mechanical and Physiological Rest, etc.,") in which the tongue was decidedly furred only on the side corresponding with carious or painful teeth; one in which the hair of the left temple was bleached by unilateral neuralgia, arising from a carious molar tooth; and one in which ulceration of the auditory canal, and enlargement of one of the cerival glands, was traced to a diseased lower molar on the same side, and subsided soon after the extraction of the tooth.

XV.—*Intense neuralgia of the eyeball and face; alteratnio of the color of the iris; carious teeth.*—Mrs. C—, aged thirty, had suffered for ten years from severe neuralgia, affecting the left eyeball and left side of the head and face; the iris of the affected eye having changed from a deep and bright hazel to a dull grey. The left lower *dens sapientiæ* and the first upper bicuspid being found badly carious, these were extracted, and the operation "was attended by a terrible paroxysm of neuralgia," but after this had subsided, the patient experienced relief for about three mnoths, when, the old pain returning, the second bicupid was found to be carious and intensely tender, and upon its removal a considerable exostosis was seen on the root. "The pain vanished with the tooth."

XVI.—*Superficial sloughing of the cheek caused by a carious tooth-stump.*—A young woman applied to Mr. Bell on account of a remarkable oval slough of the skin and cellular tissue, about the size of a shilling, just beneath the orbit. It was removed, but soon returned. On the extraction of a diseased stump on the same side of the upper jaw, the slough separated and the sore healed.

XVII.—*Ulceration of the neck from a carious dens sapientiæ.*—M. S.—, æt. twenty-two had an ulcer in the neck, behind and below the angle of the lower jaw on the right side. It had commenced more than a year before, as a painful red spot, which appeared spontaneously and had speedily assumed its present ulcerated condition, resisting all treatment. Her appearance was perfectly healthy. The right lower wisdom tooth, which was carious, with much neighboring irritation, was extracted, and in a week or ten days the sore had healed. The ulcer was quite superficial, and had no fistulous connection with the tooth.

### Direct Affections,

The portio dura of the seventh nerve, and the nerves which enter the orbit, are suppossed by Mr. Salter to be the only ones which suffer in this way.

XVIII. *Facial Paralysis, etc., from carious dens sapientiæ.*—Mrs. Barnard had frequeutly suffered from toothache without swelling in the right upper wisdom tooth, during two years. In December, 1864, an attack of violent pain set in, which gradually extended from the tooth to the front of the jaw and up to the eye; the throat on the right side was also affected, and the right arm nearly paralyzed and aching constantly. The mouth and features were drawn toward the left side, and the vision of the right eye became very dim, with dull aching behind the globe. On January 14th, the right superior *dens sapientiæ*, which had a carious cavity opening the central pulp chamber, was removed and brought with it a piece of alveolus. The fang was covered with patches of red lymp. Extraction was followed by immediate relief. On February 8th, the patient still complaining of wandering pains about the eye, and in the lower jaw of the right side, the right second lower molar, which was badly carious, was removed, and these remaining symptoms soon disappeared.

XIX. *Amaurosis from abscess of the antrum, produced by a carious tooth.*—Elza F—, æt. twenty-four, previously in good health, was suddenly attacked with violent pain about the remains of the first right upper molar tooth, the crown of which had been lost by caries. Enormous swelling of the side of the face came on rapidly; infiltration the lower lid which nearly closed the eye; protrusion of the malar bone; a thrusting over of the nose to the opposite side, and blindness of right eye. In about twenty-four hours matter pointed just below the inner canthus. This was evacuated by the lancet, and after the lapse of another twenty-four hours a fresh pointing appeared below the outer canthus, which was also punctured. After suffering much for two or three weeks she was admitted to Guy's Hospital, the enormous swelling still continuing; the eye ball protruding; great conjunctivitis; sight entirely gone; and profuse discharge of grumous pus from the fistulous orifice below the outer canthus. The fangs of the crownless first molar and a carious wisdom tooth were removed, the operation causing great purulent discharge from the opening near the eye and also from the right nostril, while the withdrawal of the molar fangs opened the floor of the antrum, and led to an abundant discharge into the mouth. The symptoms gradually subsided, and on the removal of a considerable sequestrum from the floor of the orbit, maxilla, and outer wall of the nose, all inflammatory action ceased. From time to time smaller portions of necrosed bone came away, and the patient was discharged, but kept under observation as a night nurse in the hospital. "The loss of vision of the affected eye has remained permanent, and the necrosis of bone below the orbit has produced a persistent opening into the antrum." A curious feature of this case is that the ophthalmoscope reveals perfect transparancy of the humors of the eye, and a healthy condition of the retina. The only abnormal appearance has been "extreme *anœmia of the optic nerve* at its abutment on the retina. Instead of exhibiting the ordinary yellowish-pink disc, it has presented a white, circular area, characteristic of anæmia of the optic nerve, so constantly associated with suspension of the function of vision dependent upon causes external to the globe.

XX.—*Amaurosis; antral abscess; carious molar tooth; splinter of toothpick imbedded in alveolus.*—In this case, (reported by Professor Galenzowski, in the Archives Génér- ales de Medécine, tome xxiii, p. 261, Paris' 1830,) F. P—, æt. thirty, "suddenly experienced violent pain, extending from the left temple to the eye, and that side of the face. It diminished and recurred with renewed force at intervals. About two months afterwards the pain suddenly became very intense, especially in the eye; the globe had a painful sense of protrusion. The patient now discovered that he was blind of the left eye. The pain then diminished; at the end of six months it again increased, the cheeks swelled, and some spoonfuls of sanious pus were discharged through a spontaneous opening in the lower lid; then the swelling went down, and the pain nearly vanished. The blindness remained complete. The discharge was renewed from time to time; there was no great suffering. But in the autumn and winter the pain, especially in the eye, became so severe that F. P— came to Wilna, determined to have the globe extirpated, if no other remedy could be discovered." The left eye was found quite insensible to light, and the pupil dilated; there was no other visible alteration. "The pain less severe, consisted in pricking and darting sensations in the left temple and around the eye; the discharge through the lower lid continued. The first upper molar tooth was carious; it had not caused much pain, and the toothache, when there was any, was not coincident with the pains in the temple and eye. This carious tooth was now extracted, and at the end of the fang a foreign body was found, which upon examination, proved to be a splinter of wood, probably the end of a toothpick; it was a quarter of an inch in length. The alveolar cavity communicated with the antrum, and some pus escaped from the sinus. The pain now ceased almost entirely, and on the same evening the eye became sensible of light. Vision gradu[illegible]lly improved, so that on the ninth day after the extraction of the tooth, F. P— could see as well with the left eye as with the right, after a blindness of thirteen months. On the eleventh day he left Wilna to return to his family."

XXI.—*Amaurosis caused by a carious molar tooth.*—"A gentleman, æt. about thirty-five, was attacked with intense deep-seated inflammation of the superior maxillary region and orbit, more especially the latter, involving the eye in universal congestion of its tissues. The globe was protruded, the sight completely gone, and the pupil dilated and fixed under all circumstances. Upon examination, Mr. Pollock found that there was much tenderness on pressure, extending downwards from the inner edge of the lower margin of the orbit towards the jaw on the affected side, and upon scrutinizing the condition of the mouth some carious teeth were found, which, for want of any other evident cause, he considered might be the origin of the existing mischief." The fangs of the first premolar and the first true molar teeth were extracted, the latter showing indications of great irritation about its roots. "The removal of the teeth was followed by the immediate and complete subsidence of all the inflammatory symptoms, and in about ten days all heat and redness, as well as the apparent inflammation of the eye, had vanished." The pupil gradually resumed contractillity, but the eye has remained sightless, though perfectly natural to external appearance.

XXII.—*Amaurosis; antral abscess; carious teeth.*—The case recorded by Dr. Bruck, in Casper's *Wochenschrift* for March, 1851, of a healthy man, æt. forty-five, who had suffered for many years from chronic inflammation of the mucus membrane of the nose and maxillary sinus on the left side, with frequent severe acute attacks which involved the whole maxillary bone on the side of the affected teeth, the tongue, and throat, and extending up to the scalp, which became intensely sensitive. "There was great injection of the eyelids, the globe protruded, and there was much and severe toothache. After a few days, matter, previously pent up in the antrum, burst through the left nostril, and the patient recovered from the acute symptoms, which, however, again and again recurred. The interesting point of this case is the relation of the eye to that of the antrum. The former appears to have followed and been dependent upon the latter. On the occasion of the acute attacks, the globe was protruded, as Dr. Bruck says, by the vaulting of the roof of the maxillary sinus from its distending contents, and all the structures within the orbit appear to have shared the inflammation. In an early stage of the case *vision was entirely lost, and the axis of the eye diverged.* Under treatment the symptoms of the antral affection gradually disappeared, and with their disapppearance the sight of the affected eye returned

XXIII.—*Amaurosis ; antral abscess ; carious teeth.*—Mr. Gaine, of Bath, reports (*Brit. Med. Jour.*, No. cclxi, p. 683,) the case of E. B—, æt. twenty-two, who was admitted into Bath Hospital, August 7th, 1863, on accouut of defective vision of the right eye. "There was *ptosis* of the right upper eyelid, also much swelling of the face, with an alveolar, and, as it proved, an antral abscess associated with the first right upper molar tooth, which had been broken off in an attempt to extract it." About five weeks afterwards the sight of the right eye was entirely lost. "The stumps of the broken tooth were extracted, the antrum pierced, and a large quantity of pus evacuated. The optic nerve, as seen by the ophthalmoscope, was very anæmic. When seen by Mr. Gaine on May 5th, 1864, the third nerve had recovered ; the ptosis had disappeared, and there was a fair action of the pupil. The optic nerve was still anæmic, and the right eye entirely blind.

In commenting upon these cases, Mr. Salter asks : "As regards the anæmia of the optic nerve, are we to consider it the result of mechanical obstruction to the circulation, or does it simply depend upon, and is it secondary to, suspended function ? A circumferential plastic exudation encasing the nerve might possibly strangle by pressure the circulation within it, or an interstitial deposit of lymph between the fibrillæ might effect that consequence more readily and completely. Then, on the other hand, it is well known that a suspension of the function of sight by causes external to the globe itself, even so remote as disease of the brain, will entail complete anæmia of the optic nerve. The precise relation, therefore, of the sightlessness of the eye and the anatomical condition in question must remain matter of doubt. *Med. Gazette.*

